# Longitudinal fasting blood glucose patterns and arterial stiffness risk in a population without diabetes

**DOI:** 10.1371/journal.pone.0188423

**Published:** 2017-11-20

**Authors:** Yuntao Wu, Junxing Yu, Cheng Jin, Yun Li, Jinmei Su, Guoqing Wei, Xiaoming Zheng, Jingsheng Gao, Wenyuan Gao, Shouling Wu

**Affiliations:** 1 School of Pharmaceutical Science and Technology, Tianjin University, Tianjin, China; 2 Department of Cardiology, Kailuan General Hospital, North China University of Science and Technology, Tangshan, China; 3 Graduate School, North China University of Science and Technology, Tangshan, China; 4 School of Public Health, North China University of Science and Technology, Tangshan, China; 5 Department of Rheumatology, Peking Union Medical College Hospital, Peking Union Medical College & Chinese Academy of Medical Sciences, Key Laboratory of Rheumatology and Clinical Immunology, Ministry of Education, Beijing, China; Shanghai Diabetes Institute, CHINA

## Abstract

**Objective:**

To identify long-term fasting blood glucose trajectories and to assess the association between the trajectories and the risk of arterial stiffness in individuals without diabetes.

**Methods:**

We enrolled 16,454 non-diabetic participants from Kailuan cohort. Fasting blood glucose concentrations were measured in 2006, 2008, and 2010 survey. Brachial-ankle pulse wave velocities were measured during 2011 to 2016. Multivariate regression model was used to estimate the difference of brachial-ankle pulse wave velocity levels and logistic regression was used to calculate odds ratios (ORs) and 95% confidence intervals (95%CIs) of arterial stiffness risk, according to the fasting blood glucose trajectories.

**Results:**

We identified five distinct fasting blood glucose trajectories and each of the trajectories was labeled according to its range and change over 2006–2010 survey: elevated-stable pattern (5.0% of participants), elevated-decreasing pattern (6.6%), moderate-increasing pattern (10.9%), moderate-stable pattern (59.3%), and low-stable pattern (18.2%). After adjustment for potential confounders, individuals with elevated-stable pattern had a 42.6 cm/s (95%CI: 24.7 to 60.6 cm/s) higher brachial-ankle pulse wave velocity level and a 37% (OR 1.37, 95%CI: 1.14 to 1.66) higher arterial stiffness risk, and individuals with moderate-increasing pattern had a 19.6 cm/s (95%CI: 6.9 to 32.3 cm/s) higher brachial-ankle pulse wave velocity level and a 17% (OR 1.17, 95%CI: 1.03 to 1.33) higher arterial stiffness risk, related to individuals with moderate-stable pattern. We did not find significant associations of the elevated-decreasing or low-stable patterns with arterial stiffness. Consistently, the cumulative average, variability, and increased rate of fasting blood glucose during 2006–2010 survey were significantly associated with the arterial stiffness risk.

**Conclusion:**

Discrete fasting blood glucose trajectories were associated with the arterial stiffness risk in non-diabetic individuals.

## Introduction

Arterial stiffness, a surrogate measure of vascular health, becomes a novel cardiovascular disease (CVD) biomarker[1, 2]. Brachial–ankle pulse wave velocity (baPWV) reflects organic and functional stiffness of the arterial wall and can be used to assess the arterial stiffness simply, reproducibly and noninvasively[3]. Recent studies showed that the high baPWV had a strong predictive value for the future cardiovascular risk in general population[4] and diabetes mellitus patients[5, 6].

High blood glucose concentration was associated with the high CVD and mortality risk[7, 8]. The mechanisms leaded to CVD remain unclear but might involve increased arterial stiffness [9, 10]. Some studies[11, 12] consistently showed that diabetes might increase arterial stiffness, however, the association between glucose concentration and arterial stiffness was controversial in populations without diabetes[9, 10, 13–16]. These controversial studies were limited with relatively small sample size [9, 13], selected populations (the elderly)[10, 15], or without adjustment for use of medication[13]. Furthermore, all of the studies [9, 10, 13–16] on this topic were based on a single measure of fasting blood glucose (FBG), failing to take into account the potential effect of average and change in FBG concentrations over time, however, the malignant effect of abnormal FBG might be cumulative for years before noticeable arterial stiffness was formed.

The Kailuan study, a community population based cohort study, measured comprehensive cardiovascular risk factors including FBG every two years since 2006. Successive FBG assessments over time could identify different trajectories of change in FBG as separate patterns, and provided more realistic understanding of long time trend. BaPWV was measured in 2011–2016 in a sample of the Kailuan cohort. We hypothesized that multiple FBG trajectories exist among this sample and individuals with higher levels and/or faster change of FBG trajectory would have higher arterial stiffness risk, relative to those with normal and stable FBG levels.

## Materials and methods

### Study design and population

The Kailuan study, a cohort study in Tangshan, China, was designed to investigate the risk factors of common non-communicable chronic diseases. The detail of study design and procedure was published previously [17]. In brief, 101,510 employees including the retired people of the Kailuan Company participated in the health survey, which included questionnaire assessments, clinical examination, and laboratory tests during 2006–2007 (referred to as the 2006 survey here). After that, all employees were invited to perform the health examination repeatedly biennially to update information on potential risk factors and lately diagnosed diseases.

Current study enrolled 19,563 participants with two or more FBG measurements in 2006, 2008 and 2010 survey, and with PWV measurement between 2011 and 2016. The baPWV assessments were initially designed to address various chronic diseases/conditions and their indicators, including asymptomatic polyvascular abnormalities, peripheral arterial disease, aging, and maternal health [18–21]. Participants with PWV data were younger (45.0 vs. 53.2 years), had a larger proportion of women (34.8% vs. 17.2%), relative to those without PWV data (n = 81,947).

In the current analyses, we excluded 3051(16.6%) participants with diabetes in or prior to 2010 survey. Because individuals with atrial fibrillation or flutter were difficult to give a precise PWV reading, we further excluded 58 participants with atrial fibrillation or flutter. Finally, a total of 16,454 participants were included in the statistical analyses. This study was performed according to the guidelines of Helsinki Declaration and was approved by the Ethics Committees of the Kailuan General Hospital. The authors obtained written informed consent from the participants before they were enrolled into the study.

### Assessment of FBG, FBG trajectories, and diabetes

Fasting blood samples were collected in the morning after an 8- to 12-hour overnight fast and transfused into vacuum tubes containing EDTA (Ethylene Diamine Tetra Acetic acid). Plasma was separated from blood immediately and kept at **4°C.** Blood glucose concentration was measured in 4 hours after plasma separation by an auto analyzer (Hitachi 747; Hitachi, Tokyo) with the hexokinase/ glucose-6-phosphate dehydrogenase method [22] in 2006, 2008, 2010, 2012, and 2014 survey, once per survey. The coefficient of variation using blind quality control specimens was <2.0%. Diabetes was defined as a self-reported physician-diagnosis history, currently treated with insulin or oral hypoglycemic agents, or a FBG concentration 7.0 mmol/L or higher.

FBG trajectories during 2006, 2008, and 2010 survey were identified with latent mixture modeling by the PROC Traj procedure[23, 24]. We used the censored normal model for FBG, a continuous variable. Model fit was assessed using the Bayesian Information Criterion (BIC). We fitted models with 5 trajectories, and then compared the BIC to those with 4, 3, 2, and 1 trajectory respectively. After the model with 5 trajectories was identified fit best, we then compared the model with different functional forms. Quadratic and linear terms were considered and evaluated based on their significance level (p<0.05), starting with the highest polynomial. In our final model, we had 4 trajectories with quadratic order terms and 1 trajectory with linear order terms.

### Assessment of baPWV and arterial stiffness

BaPWV was assessed by BP-203 RPE III networked arteriosclerosis detection device [Omron health medical (China) Co., LTD] following the manufacture’s recommendations, which was performed by specially trained nurses between 7am and 9am on the examination day. Smoking and drinking of caffeinated drinks or alcohol were prohibited for at least 3 hours, and exercise was prohibited for at least 30 minutes before baPWV measurements. After being seated for at least 5 minutes in a room with temperature controlled between 22 and 25°C, participants were asked to lay down on the examine couch in a supine and keep quite during the measurement. Cuffs were wrapped on both arms and ankles. The lower edge of the arm cuffs were positioned 2–3 cm above the transverse striation of cubital fossa, while the lower edge of the ankle cuffs were positioned 1–2 cm above the superior aspect of medial malleolus. The electrocardiogram electrodes were placed on both wrists and a microphone for detecting heart sounds was placed on the left edge of the sternum. We used the mean of baPWV in analyses because the right and the left baPWV are significantly correlated. The value of baPWV equal or higher than 1400 cm/s was consider as arterial stiffness since previous studies suggested that a cutoff value of 1400 cm/s could be used for screening participants with high cardiovascular risks[5, 25], and was recommended in the guideline for CVD presentation in China[26].

### Assessment of covariates

Information on potential covariates was collected in 2006 survey and updated biennially thereafter, as detailed elsewhere [22]. In brief, information on age, gender, smoking, alcohol intake, physical activity, average monthly income, education level, past medical history (e.g., hypertension, diabetes, atrial fibrillation, CVD, and active treatment such as hypoglycemic, antihypertensive, aspirin, or lipid regulating medications agent) was collected via questionnaire. Diagnosis of atrial fibrillation or flutter was based on 12-lead electrocardiogram or self-reported physician-diagnosis history. Height, weight, systolic blood pressure (SBP) and diastolic blood pressure (DBP) were assessed by trained field-workers (i.e., nurses) during the surveys. Body mass index (BMI) was calculated as weight in kilogram/ height^2^ in meters. Total cholesterol, triglycerides, high-density lipoprotein cholesterol (HDL-C), low-density lipoprotein cholesterol (LDL-C), and high sensitive C-reactive protein (hs-CRP) were assessed by auto analyzer (Hitachi 747; Hitachi, Tokyo, Japan) at the central laboratory of Kailuan hospital. Estimated glomerular filtration rate (eGFR) was calculated by the Chronic Kidney Disease Epidemiology Collaboration creatinine equation[27].

### Statistical analyses

Statistical analyses were completed by using SAS version 9.3 (SAS Institute, Inc, Cary, NC). As missing data for covariates was little (ranged from 0 to 2.68%), we applied median imputation to deal with the missing continue variable, and add an extra category for the categorical variable to indicate missing. We used multivariate regression models to estimate the association between five FBG trajectories and baPWV levels. The moderate-stable pattern was set as reference group for it had the most participants (59.3%). We selected potential confounders a priori based on available literature and assessment of a causal diagram. Results were adjusted for potential cofounders, including age, sex, use of antihypertensive, aspirin, and lipid-lowering medications, smoke status, alcohol intake, education, physical activity, sodium intake, family income, SBP, DBP, BMI, eGFR, and blood concentration of HDL-C, LDL-C, triglycerides, and hs-CRP. Because blood pressure might be the middle part of the causal chain of blood glucose and baPWV, we did a model without adjustment for SBP and DBP.

To examine whether the potential association between high FBG and increased baPWV was due to the higher likelihood of development to diabetes, we conducted a sensitivity analysis by excluding individuals who developed to diabetes during 2010 to 2016. To explore whether the potential association between FBG and baPWV was confounded by medications, we conducted a sensitivity analysis by excluding the participants who used aspirin, antihypertensive, or lipid regulating medications. We also restricted the analysis to 16,062 participants with complete data on all covariates. We examined potential interactions of FBG trajectories with age (<50, vs. ≥50 years), sex, blood pressure status (hypertension, and non-hypertension), and health status (health or non-health). We included multiplicative terms in the regression models, with adjustment for other potential confounders. The correlations between FBG trajectories and bpPWV were also analyzed after stratifying by these variables.

As secondary exposures, we also calculated cumulative average FBG (average of all available FBG values during 2006–2010 survey), annual FBG increase rate (the slope of the simple linear regression model in which a FBG value was the response variable and follow-up duration was the independent variable) and FBG variability (as assessed by standard deviation) during 2006–2010 survey; their associations with bpPWV levels were examined. We also used logistic regression to test differences in arterial stiffness prevalence across categories and to calculate odds ratios (ORs) and 95% confidence intervals (CIs), after adjustment for aforementioned covariates.

## Results

**Fig 1:**
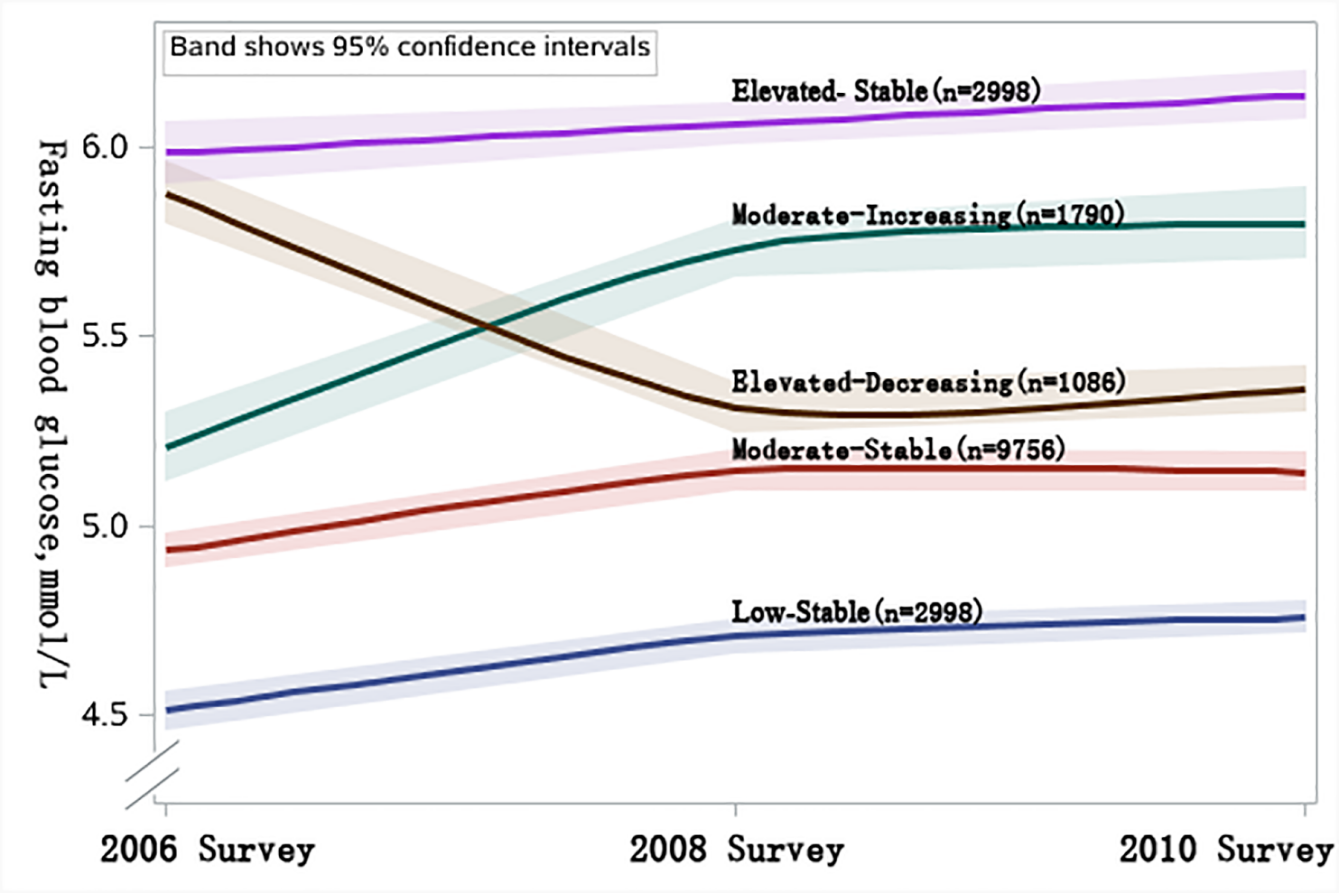
Trajectory of fasting blood glucose trajectory patterns during 2006 to 2010 Survey, among 16,454 Kailuan participants.

Based on the FBG concentrations and change patterns during 2006 to 2010 survey, we categorized the study population into five trajectories (Fig 1): 824 (5.0%) participants who had elevated FBG concentrations (mean FBG concentrations increased from 6.12 to 6.23mmol/L; referred to as “elevated-stable pattern”); 1086 (6.6%) participants who started with elevated concentrations and experienced a decrease (mean FBG concentrations decreased from 6.15 to 5.36mmol/L; referred to as “elevated-decreasing pattern”); 1790 (10.9%) participants who started with moderate concentrations and experienced an increase (mean FBG concentrations increased from 5.23 to 5.99mmol/L; referred to as “moderate-increasing pattern”); 9756 (59.3%) participants who maintained moderate FBG concentrations (mean FBG concentrations ranged from 4.96 to 5.16mmol/L; referred to as “moderate-stable pattern”); and 2998 (18.2%) participants who maintained low FBG concentrations(mean FBGs ranged from 4.35 to 4.62mmol/L; referred to as “low-stable pattern”). Individuals with elevated-stable pattern were more likely to be men, older, using antihypertensive medications, having concurrent cardiovascular risk factors, having lower education and income (Table 1).

**Table 1:** Clinical and biochemical characteristics according to the fasting blood glucose trajectories during 2006 to 2010 survey, among 16,454 Kailuan participants.

	Low-Stable	Moderate-Stable	Moderate-Increasing	Elevated-Decreasing	Elevated- Stable	*P*^§^_-value_
**N (%)**	2998(18.2)	9756(59.3)	1790(10.9)	1086(6.6)	824(5.0)	
**Age, year**	43.2±12.8*	44.7±11.9	47.9±11.2*	44.9±10.2	49.1±10.9*	< .0001
**Women, %**	1481(49.4)*	3446(35.3)	449(25.1)*	240(22.1)*	140(17.0)*	< .0001
**Smoking status, %**						
**Never**	2079(69.3)*	6212(63.7)	1020(57.0)*	597(55.0)*	401(48.7)*	< .0001
**Past**	106(3.5)*	452(4.6)	106(5.9)*	74(6.8)*	64(7.8)*	
**Current**	813(27.1)*	3087(31.6)	662(37.0)*	415(38.2)*	356(43.2)*	
**Alcohol intake, %**						
**Never**	1930(64.4)*	5536(56.7)	909(50.8)*	518(47.7)*	346(42.0)*	< .0001
**Past**	59(2.0)*	235(2.4)	63(3.5)*	29(2.7)*	23(2.8)*	
**Current**	1009(33.7)*	3980(40.8)	816(45.6)*	539(49.6)*	452(54.9)*	
**Physical activity, %**						
**Never**	295(9.8)	1074(11.0)	238(13.3)*	154(14.2)*	109(13.2)*	< .0001
**1–2 times/week**	2300(76.7)	7481(76.7)	1305(72.9)*	799(73.6)*	573(69.5)*	
**3+ times/week**	401(13.4)	1186(12.2)	244(13.6)*	133(12.2)*	137(16.6)*	
**Salt intake, %**						
**≥10 gram/day**	298(9.9)	1009(10.3)	177(9.9)	98(9.0)	88(10.7)*	0.004
**6–9 gram/day**	2381(79.4)	7635(78.3)	1392(77.8)	854(78.6)	613(74.4)*	
**<6 gram/day**	317(10.6)	1097(11.2)	218(12.2)	134(12.3)	118(14.3)*	
**Education, %**						
**Illiteracy or elementary school**	121(4.0)*	446(4.6)	133(7.4)*	72(6.6)*	63(7.6)*	< .0001
**Middle school**	2278(76.0)*	7716(79.1)	1430(79.9)*	862(79.4)*	664(80.6)*	
**College /university**	596(19.9)*	1577(16.2)	224(12.5)*	152(14.0)*	92(11.2)*	
**Average income, %**						
**<¥500/month**	741(24.7)*	2476(25.4)	517(28.9)*	320(29.5)*	260(31.6)*	< .0001
**¥500 to ¥2999/ month**	1870(62.4)*	6139(62.9)	1068(59.7)*	653(60.1)*	471(57.2)*	< .0001
**≧¥3000/ month**	376(12.5)*	1070(11.0)	190(10.6)*	111(10.2)*	83(10.1)*	< .0001
**Use of antihypertensive agent, %**	337(11.2)*	1361(14.0)	400(22.3)*	171(15.7)	195(23.7)*	< .0001
**Use of lipid-lowering agents, %**	48(1.60)	143(1.47)	52(2.91)*	26(2.39)*	17(2.06)*	< .0001
**Use of Aspirin, %**	32(1.07)	111(1.14)	19(1.06)	15(1.38)	13(1.58)	0.71
**Cardiovascular Disease**	42(1.40)*	209(2.14)	40(2.23)	19(1.75)	20(2.43)	0.08
**BMI, Kg/m**^**2**^ ^**†**^	23.8±3.2*	24.6±3.1	25.7±3.2*	25.0±2.9*	26.0±3.0*	< .0001
**hs-CRP**^**†**^^**‡**^**, mg/mL**	0.83(0.45–1.62)	0.87(0.47–1.63)	1.04(0.56–1.94) *	0.94(0.51–1.64)	1.11(0.63–2.01) *	< .0001
**TC**^**†**^**, mmol/L**	4.72±0.84*	4.88±0.85	5.12±0.91*	5.05±0.83*	5.21±0.86*	< .0001
**TG**^**†**^**, mmol/L**	1.35±0.88*	1.58±1.09	1.91±1.45*	1.85±1.45*	2.01±1.40*	< .0001
**HDL-C**^**†**^**, mmol/L**	1.57±0.32*	1.54±0.31	1.54±0.33	1.56±0.30	1.55±0.39	< .0001
**LDL-C**^**†**^**, mmol/L**	2.29±0.64*	2.48±0.60	2.61±0.62*	2.66±0.57*	2.70±0.63*	< .0001
**SBP**^**†**^**, mmHg**	120±16*	125±16	131±16*	128±16*	135±16*	< .0001
**DBP**^**†**^**, mmHg**	79±9*	82±9	85±9*	84±9*	87±9*	< .0001
**eGFR**^**†**^**, mL/min/1.73m**^**2**^	91.8±15.8*	90.2±16.6	90.8±15.9	91.6±17.7*	91.2±16.3	< .0001

* indicate p< 0.05, comparing with the moderate-stable trajectory.

^§^ Continuous variables satisfied normally distributed were compared using one-way ANOVA; multiple comparison tests were performed by Dunnett’s two-tailed t test. Hs-CRP was compared using nonparametric tests (Kruskal-Wallis test). Categorical variables are compared using Chi-square test.

^†^ Average concentrations based on three measurements in 2006, 2008, and 2010 survey

^‡^ Median (25th percentile - 75th percentile)

Abbreviations: TC, Total cholesterol; TG, triglycerides; HDL-C, high-density lipoprotein cholesterol; LDL-C, low-density lipoprotein cholesterol; hs-CRP, high sensitive C-reactive protein; BMI, body mass index; SBP, systolic blood pressure; DBP, diastolic blood pressure; eGFR, estimated glomerular filtration rate.

Although adjustment for average SBP and DBP during 2006–2010 survey led to a great attenuation on the association strength between FBG trajectories and baPWV, the elevated–stable and moderate-increasing patterns remain have significant higher baPWV, comparing with the moderate-stable pattern. Individuals with the elevated-stable pattern had a significantly 42.6 cm/s (95%CI, 24.7 to 60.6 cm/s) higher baPWV; the low-stable trajectory had a -2.6cm/s (95%CI, -13.0 to 7.7 cm/s) lower baPWV without statistical significance. Of note, although individuals with the moderate-increasing and elevated-decreasing FBG patterns had similar average FBG (5.69±0.16, and 5.62±0.14 mmol/L, respectively) during 2006–2010, individuals with the moderate–increasing FBG pattern had a 19.6 cm/s (95%CI, 6.9 to 32.3 cm/s) higher baPWV, whereas individuals with the elevated-decreasing FBG pattern had a 4.5 cm/s (95%CI, -11.1 to 20.2 cm/s) higher baPWV without reached statistical significance, relative to individuals with the moderate-stable FBG trajectory (Table 2).

**Table 2:** Mean difference and 95% confidence intervals of brachial-ankle pulse wave velocity according to the fasting blood glucose trajectory patterns, among 16,454 Kailuan participants.

	Low-Stable	Moderate-Stable	Moderate-Increasing	Elevated-Decreasing	Elevated-Stable
**n (%)**	2998(18.2)	9756(59.3)	1790(10.9)	1086(6.6)	824(5.0)
**Age and sex adjusted**	-22.9(-34.2 to -11.6)	0(reference)	50.9(37.0 to 64.8)	23.8(6.50 to 41.1)	95.2(75.6 to 114)
**Multiple adjusted** ^**†**^	-16.2(-27.3 to -5.10)	0(reference)	34.5(20.8 to 48.2)	13.3(-3.50 to 30.2)	75.9(56.7 to 95.2)
**Advanced adjusted BP** ^**‡**^	-2.60(-13.0 to 7.70)	0(reference)	19.6(6.90 to 32.3)	4.50(-11.1 to 20.2)	42.6(24.7 to 60.6)
**Sensitively analyses**					
**Excluded 1080 incident diabetes during 2010–2015**^**‡**^	-1.70(-11.9 to 8.50)	0(reference)	6.00(-7.30 to 19.4)	3.00(-13.0 to 19.1)	22.2(1.60 to 42.8)
**Excluded 335 participants with CVD history prior to 2010**^**‡**^	-3.90(-14.2 to 6.50)	0(reference)	18.9(6.10 to 31.7)	3.30(-12.4 to 19.0)	39.3(21.3 to 57.4)
**Excluded 2662 participants use of medications** ^‡^^*^	-0.60(-11.1 to 10.0)	0(reference)	16.3(2.60 to 30.0)	6.90(-9.40 to 23.2)	48.0(28.5 to 67.6)
**Excluded 450 participants with missing data**^**‡**^	-2.20(-12.7 to 8.20)	0(reference)	20.5(7.60 to 33.3)	5.40(-10.4 to 21.2)	43.2(24.9 to 61.4)

^†^ Model adjusted for age (year), sex, smoke status (current, past, or never), alcohol intake (current, past, or never), education (illiteracy/elementary school, middle school, or college/university), average monthly income of each family member (<500, 500–2999, or ≥3000¥), salt intake (≥10.0, 6.0–9.9, or <6.0 gram/day), physical activity(never, 1–2 times/week, or 3+ times/week), updated use of antihypertensive, aspirin, lipid-lowering medications (yes/no for each), and cumulative average body mass index (kg/m^2^), triglycerides(mmol/L), high-density lipoprotein cholesterol(mmol/L), low-density lipoprotein cholesterol(mmol/L), high sensitive C-reactive protein (mg/L), and estimated glomerular filtration rate(mL/min/1.73m^2^).

^‡^ Model included variables in model^†^ and further adjusted for systolic blood pressure (mmHg) and diastolic blood pressure (mmHg).

* Excluding the participants who used aspirin, antihypertensive, or lipid regulating medications.

Similar results were observed when we excluded 1080 incident diabetes from 2010–2014 survey, excluded 335 participants with CVD history in or prior to 2010 survey, excluded 2662 participants used aspirin, antihypertensive, or lipid regulating medications **or** restricted the analyses to 16,062 participants with complete data (Table 2). We observed a significant interaction between FBG trajectories and age (P interaction<0.001) but not between FBG trajectories and sex (P interaction = 0.38), hypertension status (P interaction = 0.85), or heath status (P interaction = 0.69). The association between elevated-stable pattern and PWV was stronger among individuals <50 years of age, relative to the elderly (S1 Table).

The FBG trajectory was a strong predictor for arterial stiffness (baPWV ≥1400 cm/s), adjusted odds ratio was 1.37(95%CI, 1.14 to 1.66) for the elevated-stable pattern, and 1.17(95%CI, 1.03 to 1.33) for the moderate–increasing FBG pattern relate to the modern-stable pattern (Table 3). Furthermore, we further found that both the higher average FBG and FBG variability between 2006 and 2010 survey were associated with the higher arterial stiffness risk (Table 4).

**Table 3:** Multivariable adjusted odds ratios and 95% confidence intervals (95% CIs) of arterial stiffness, according to the fasting blood glucose trajectory patterns, among 16,454 Kailuan participants.

	Low-Stable	Moderate-Stable	Moderate-Increasing	Elevated-Decreasing	Elevated-Stable
**# arterial stiffness (prevalence)**	1237(41.3)	4901(50.2)	1185(66.2)	607(55.9)	619(75.1)
**Age and sex adjusted**	0.84(0.77 to 0.93)	1(reference)	1.50(1.34 to 1.69)	1.10(0.96 to 1.26)	2.04(1.71 to 2.43)
**Multiple adjusted** ^**†**^	0.91(0.82 to 1.00)	1(reference)	1.33(1.18 to 1.50)	1.00(0.87 to 1.15)	1.74(1.45 to 2.08)
**Advanced adjusted BP**^**†**^^**‡**^	1.01(0.91 to 1.12)	1(reference)	1.17(1.03 to 1.33)	0.93(0.80 to 1.08)	1.37(1.14 to 1.66)
**Sensitively analyses**					
**Excluded 1080 incident diabetes during 2010–2015**^**‡**^	1.02(0.92 to 1.14)	1(reference)	1.10(0.96 to 1.27)	0.90(0.77 to 1.06)	1.23(0.99 to 1.53)
**Excluded 335 participants with** **CVD history prior to 2010**^**‡**^	1.01(0.91 to 1.12)	1(reference)	1.17(1.03 to 1.33)	0.93(0.80 to 1.08)	1.35(1.12 to 1.64)
**Excluded 2662 participants** **use of medications** ^‡^^*^	1.01(0.90 to 1.12)	1(reference)	1.16(1.01 to 1.33)	0.92(0.78 to 1.08)	1.38(1.12 to 1.69)
**Excluded 450 participants** **with missing data**^**‡**^	1.02(0.92 to 1.13)	1(reference)	1.20(1.06 to 1.37)	0.95(0.82 to 1.11)	1.36(1.12 to 1.65)

^†^ Model adjusted for age (year), sex, smoke status (current, past, or never), alcohol intake (current, past, or never), education (illiteracy/elementary school, middle school, or college/university), average monthly income of each family member (<500, 500–2999, or ≥3000¥), salt intake (≥10.0, 6.0–9.9, or <6.0 gram/day), physical activity(never, 1–2 times/week, or 3+ times/week), updated use of antihypertensive, aspirin, lipid-lowering medications (yes/no for each), and cumulative average body mass index (kg/m^2^), triglycerides(mmol/L), high-density lipoprotein cholesterol(mmol/L), low-density lipoprotein cholesterol(mmol/L), high sensitive C-reactive protein (mg/L), and estimated glomerular filtration rate(mL/min/1.73m^2^).

^‡^ Model included variables in model^†^ and further adjusted for systolic blood pressure (mmHg) and diastolic blood pressure (mmHg).

* Excluding the participants who used aspirin, antihypertensive, or lipid regulating medications

**Table 4:** Mean difference and 95% confidence intervals of brachial-ankle pulse wave velocity, according to the cumulative average, increase rate, and variability of fasting blood glucose from 2006 to 2010 survey.

	Fasting blood glucose					Trend^†^
	**Average fasting blood glucose during 2006 to 2010 survey**					
**Range, mmol/L**	3.36 to 4.73	4.74 to 4.99	5.00 to 5.22	5.23 to 5.52	5.53 to 6.95	
**Mean difference**^**‡**^	0(reference)	-0.20(-13.2 to 12.7)	12.0(-1.10 to 25.0)	23.3(10.0 to 36.5)	48.0(34.4 to 61.6)	41.7(31.2 to 52.2)
	**Annual increase rate of fasting blood glucose during 2006 to 2010 survey**					
**Range, mmol/L**	-1.35 to -0.11	-0.10 to 0.00	0.01 to 0.09	0.10 to 0.20	0.21 to 1.36	
**Mean difference**^**‡**^	0(reference)	1.30(-11.7 to 14.3)	1.70(-11.3 to 14.6)	8.70(-4.30 to 21.7)	17.6(4.60 to 30.6)	15.8(-2.10 to 33.7)
	**Variability of fasting blood glucose during 2006 to 2010 survey**					
**Range, mmol/L**	0.00 to 0.19	0.20 to 0.31	0.32 to 0.44	0.45 to 0.63	0.64 to 2.57	
**Mean difference**^**‡**^	0(reference)	-0.20(-13.2 to 12.7)	12.0(-1.10 to 25.0)	23.3(10.0 to 36.5)	48.0(34.4 to 61.6)	36.8(11.8 to 61.7)

^†^ Linear trends were tested for significance by assigning each participant the median value in each category to create a continuous variable.

^‡^ Model adjusted for age (year), sex, smoke status (current, past, or never), alcohol intake (current, past, or never), education (illiteracy/elementary school, middle school, or college/university), average monthly income of each family member (<500, 500–2999, or ≥3000¥), salt intake (≥10.0, 6.0–9.9, or <6.0 gram/day), physical activity(never, 1–2 times/week, or 3+ times/week), updated use of antihypertensive, aspirin, lipid-lowering medications (yes/no for each), and cumulative average body mass index (kg/m^2^), triglycerides(mmol/L), high-density lipoprotein cholesterol(mmol/L), low-density lipoprotein cholesterol(mmol/L), high sensitive C-reactive protein (mg/L), and estimated glomerular filtration rate(mL/min/1.73m^2^).

## Discussions

In the current study, we observed five heterogeneous FBG trajectories in 16,454 non-diabetes participants in Kailuan sub-cohort with over a 4-years follow up. Non-diabetes participants with t elevated-stable or moderate–increasing FBG trajectory had higher baPWV level and higher arterial stiffness risk, relative to those with low FBG trajectories. We also observed that the high average, variability, and increment of FBG during 2006–2010 survey were significantly associated with high baPWV level. These suggested that even if the FBG level was not up to the diagnostic criteria for diabetes, physicians should paid attention to control the FBG level in the ideal range and to reduce the risk of arterial stiffness.

The trajectory method has the advantage of combination the average, variability, and the direction of variability to predict the future risk. Previous studies generated inconsistent results regarding to the association between impaired fasting glucose (IFG) [28] and arterial stiffness risk. Julio A et al[13] suggested that diabetes, but not IFG, was associated with the higher risk of large artery stiffness. In contrast, we discovered that the elevated-stable pattern participants with FBG levels in the range of IFG had the greatest risk of arterial stiffness even after we excluded those who developed diabetes during 2010–2014 survey, suggesting that the observed association might not be totally explained by the development of diabetes. The Julio A et al [13] study, had relative small sample (n = 1654), did not adjust for antihypertensive therapy, and based on a single FBG assessment, which could underestimate the true association between IFG and arterial stiffness risk[29].

Our results were in accord with the results of previous studies on the relation between FBG and arterial stiffness. Wang J et al. [30]reported that the risk of arterial stiffness in participant with impaired FBG increased by 82% compared to those with normal FBG. Ronald et al.[10] showed that impaired glucose metabolism was associated with both decreased dispensability and compliance of the artery which was measured by ultrasound. Young-Hoon Lee et al. [15] reported high-normal HbA1c level was independently associated with the risk of arterial stiffness in participants without diabetes. Some studies [16, 31] also reported a positive relationship between HbA1c and carotid intima-media thickness in the population without diabetes.

Several biological mechanisms could explain the observed association between the high FBG and arterial stiffness risk, even if the FBG concentration did not reached the diagnosis of diabetes. First, high level of FBG might induce endothelial cell apoptosis, increase the expression of intercellular adhesion molecule E and interleukin-6, and then induce vascular endothelial dysfunction[32]. Moreover, high blood glucose might make the polyether polyol, protein kinase-C and the pentose phosphate pathway strengthened, vascular endothelial cell damaged, and endothelial cells apoptosis accelerated[33]. Finally, high level of FBG would also induce the proliferation of vascular smooth muscle cells, promote its transformation from contraction to synthesis, and accelerate the arterial stiffness[34].

In our study, although the moderate-increasing and elevated-decreasing FBG patterns had similar average FBG concentration during 2006–2010 survey, individuals with the moderate-increasing FBG pattern had a significantly higher baPWV level. This result indicated that changes of FBG level had an impact on the risk of arterial stiffness. This notion is further supported by the observations that increment of FBG during 2006–2010 survey was significantly associated with arterial stiffness risk. These findings suggest that regularly monitoring FBG should make sense for public health and clinical practice. Given lifestyle intervention could effectively lower glucose concentrations and IFG were generally not been treated although they may experience high future CVD risk [35, 36], monitoring FBG among individuals with a glucose level in the range of IFG is particularly important.

Our study has several limitations. Oral glucose tolerance testing and HbA1C concentrations were not available in the Kailuan study and some diabetes cases could be thus undiagnosed. However, we identified diabetes cases based on both FBG concentrations and self-report physician diagnosis. Some recently studies reported HbA1c associated with arterial stiffness [15, 16] was persisted with our result. Secondly, although smoking and drinking of caffeinated drinks or alcohol were prohibited for at least 3 hours and exercise was prohibited for at least 30 minutes before baPWV measurements, these potential confounders might have a longer lasting action on baPWV, some variables such as daily intake of total calories, carbohydrate intake did not adjusted due to lack of data, would have some certain impact on the outcome of the study, but we have adjusted for the most important confounders, in order to minimize the impact of residual confounding factors. Third, the current study enrolled only the Chinese occupational population living in the Kailuan community and the trajectories identified in this population thus may not be generalizable to other populations. However, the homogeneous nature of our cohort could help to reduce potential confounding due to racial and healthcare disparities, and therefore, enhanced internal validity, which is a prerequisite for the generalizability. Lastly, we measured FBG concentrations in 2006, 2008, and 2010 survey, but baPWV was measured during 2011 to 2016. Although we found the moderate-increasing and elevated-stable FBG trajectories were associated with the higher arterial stiffness risk in non-diabetic individuals, future research needs to explore the relationship between FBG trajectories and the risk of developing higher baPWV.

## Conclusions

We identified five discrete FBG trajectories and found that these patterns were significantly associated with the baPWV level and arterial stiffness risk in individuals without diabetes. These observations suggest that long-term trajectories of FBG may be important for risk prediction of arterial stiffness and possibly CVD. Further studies conducted in populations without arterial stiffness at baseline are warranted to replicate our findings.

## Supporting information

S1 Table Mean difference and 95% confidence intervals of baPWV by age, sex, and hypertension statues, according to the fasting blood glucose trajectory patterns, among 16,454 Kailuan participants. (DOCX)

S1 File PLOS ONE clinical studies checklist. (DOCX)

S2 File STROBE statement. (DOCX)

S3 File Dataset. (RAR)
