# Supplementary material for: Longitudinal fasting blood glucose patterns and arterial stiffness risk in a population without diabetes
Source: PLoS One. 2017 Nov 20;12(11):e0188423. doi: 10.1371/journal.pone.0188423 (PMC5695806; doi:10.1371/journal.pone.0188423)
Supplement: S1 Table — (DOCX) [file pone.0188423.s001.docx]

**S1 Table. Mean difference and 95% confidence intervals of baPWV by age, sex, and hypertension statues, according to the fasting blood glucose trajectory patterns, among 16,454 Kailuan participants**

|  | **Low-Stable** | **Moderate-Stable** | **Moderate-Increasing** | **Elevated-Decreasing** | **Elevated- Stable** | **P _for interaction_** |
| --- | --- | --- | --- | --- | --- | --- |
| **Age < 50 years** |  |  |  |  |  |  |
| **n(%)** | 2263(19.3) | 7055(60.3) | 1105(9.4) | 802(6.9) | 478(4.1) |  |
| **Mean difference^†^** | -0.8(-11.0 to 9.3) | 0(reference) | 20.9(7.4 to 34.4) | 25.4(10.0 to 40.9) | 56.8(37.0 to 76.6) | <0.001 |
| **Age≧50 years** |  |  |  |  |  |  |
| **n(%)** | 735(15.5) | 2701(56.9) | 685(14.4) | 284(6.0) | 346(7.3) |  |
| **Mean difference^†^** | -26.2(-52.2 to -0.2) | 0(reference) | 31.8(5.0 to 58.5) | -18.0(-56.9 to 20.9) | 49.9(14.2 to 85.6) |  |
| **Women** |  |  |  |  |  |  |
| **n(%)** | 1481(25.7) | 3446(59.9) | 449(7.8) | 240(4.2) | 140(2.4) |  |
| **Mean difference^†^** | -5.4(-19.0 to 8.2) | 0(reference) | 26.0(4.0 to 48.0) | 12.5(-16.3 to 41.3) | 23.6(-14.0 to 61.3) | 0.38 |
| **Men** |  |  |  |  |  |  |
| **n(%)** | 1517(14.2) | 6310(59.0) | 1341(12.5) | 846(7.9) | 684(6.4) |  |
| **Mean difference^†^** | 3.0(-11.8 to 17.8) | 0(reference) | 19.7(4.1 to 35.3) | 3.6(-15.2 to 22.5) | 52.8(31.9 to 73.8) |  |
| **Hypertension** |  |  |  |  |  |  |
| **n(%)** | 578(11.8) | 2740(56.0) | 773(15.8) | 376(7.7) | 427(8.7) |  |
| **Mean difference^†^** | -10.9(-38.1 to 16.4) | 0(reference) | 24.1(-0.1 to 48.4) | -7.0(-39.5 to 25.5) | 56.4(25.4 to 87.5) | 0.85 |
| **Non-hypertension** |  |  |  |  |  |  |
| **n(%)** | 2420(20.9) | 7016(60.7) | 1017(8.8) | 710(6.1) | 397(3.4) |  |
| **Mean difference^†^** | 1.3(-9.2 to 11.7) | 0(reference) | 18.3(3.6 to 33.0) | 9.8(-7.4 to 27.0) | 31.0(8.4 to 53.6) |  |
| **Health** |  |  |  |  |  |  |
| **n(%)** | 688(12.2) | 3140(55.9) | 882(15.7) | 436(7.8) | 475(8.5) |  |
| **Mean difference^†^** | -10.0(-34.5 to 14.4) | 0(reference) | 19.1(-3.1 to 41.3) | -5.7(-35.2 to 23.9) | 53.8(25.2 to 82.5) | 0.69 |
| **Non-health*** |  |  |  |  |  |  |
| **n(%)** | 2310(21.3) | 6616(61.1) | 908(8.4) | 650(6.0) | 349(3.2) |  |
| **Mean difference^†^** | 0.9(-9.7 to 11.5) | 0(reference) | 21.7(6.3 to 37.0) | 12.8(-5.0 to 30.6) | 31.2(7.3 to 55.2) |  |

^†^ Model adjusted for age (year), sex, smoke status (current, past, or never), alcohol intake (current, past, or never), education (illiteracy/elementary school, middle school, or college/university), average monthly income of each family member (<500, 500-2999, or ≥3000¥), salt intake (≥10.0, 6.0-9.9, or <6.0 gram/day), physical activity(never, 1-2 times/week, or 3+ times/week), updated use of antihypertensive, aspirin, lipid-lowering medications (yes/no for each), and cumulative average body mass index (kg/m^2^), triglycerides(mmol/L), high-density lipoprotein cholesterol(mmol/L), low-density lipoprotein cholesterol(mmol/L), high sensitive C-reactive protein (mg/L) , and estimated glomerular filtration rate(mL/min/1.73m^2^).

* Non-health included self-reported physician-diagnosis history of hypertension, cardiovascular disease, cancer; systolic blood pressure ≥140 mmHg, diastolic blood pressure ≥90 mmHg; or total cholesterol ≥6.22mmol/L at 2010 survey
